# Factors affecting the integration of tobacco cessation services for TB patients

**DOI:** 10.5588/ijtldopen.25.0587

**Published:** 2026-02-11

**Authors:** B. Loabile, D. Ahmed, O. Nteba, B. Tsima, B. Kgwaadira, S. Schriger, M. Mosepele, F.K. Barg, R.A. Schnoll, R. Gross

**Affiliations:** 1 Division of Infectious Diseases, Department of Medicine, Perelman School of Medicine, University of Pennsylvania, Philadelphia, PA, USA;; 2 Department of Biostatistics, Epidemiology, and Informatics, Perelman School of Medicine, University of Pennsylvania, Philadelphia, PA, USA;; 3 Botswana University of Pennsylvania Partnership (BUP), Gaborone, Botswana;; 4 Department of Family Medicine, University of Botswana, Gaborone, Botswana;; 5 Botswana-University of Maryland School of Medicine Health Initiative (BUMMHI), Gaborone, Botswana;; 6 Department of Psychology, School of Arts and Sciences, University of Pennsylvania, Philadelphia, PA, USA;; 7 Department of Internal Medicine, University of Botswana, Gaborone, Botswana;; 8 Department of Family Medicine and Community Health, Perelman School of Medicine, University of Pennsylvania, Philadelphia, PA, USA;; 9 Department of Psychiatry and Abramson Cancer Center, Perelman School of Medicine, University of Pennsylvania, Philadelphia, PA, USA.

**Keywords:** tuberculosis, Botswana, HIV, health care integration, smoking, low- and middle-income countries

## Abstract

**BACKGROUND:**

Tobacco smoking remains a serious health threat, especially for patients with TB and HIV. Cessation services may be apt for integration into TB and HIV clinics, particularly in lower- and middle-income countries, where these services often have robust structures. We aimed to identify barriers and facilitators to the integration of smoking cessation within health care services for people with HIV on TB treatment in Botswana.

**METHODS:**

Using the Consolidated Framework for Implementation Research 2.0, we conducted a convergent parallel mixed-methods study collecting demographic data on current TB patients and semi-structured interviews with patients and providers.

**RESULTS:**

We identified four key themes on programme implementation: 1) Tobacco knowledge is necessary but insufficient to facilitate smoking cessation; 2) Providers lack skill to provide cessation services and are apprehensive about interactions with TB patients; 3) An intervention would be desirable but will require additional infrastructure and cannot exclude other non-TB populations; and 4) Leveraging non-governmental implementation partners would be an asset but may also impede programme longevity.

**CONCLUSION:**

Novel approaches are needed to overcome key barriers to the integration of smoking cessation treatments for this important subgroup of smokers, including developing a comprehensive tobacco treatment programme that can extend beyond education and provide critical skills for providers.

Over 80% of tobacco users live in low- and middle-income countries (LMICs),^1^ and smoking is a major cause of adverse health outcomes for the population.^2^ Smoking and secondhand smoke increase the risk of TB,^3^ poor TB outcomes,^4^ and TB transmission.^5^ In sub-Saharan Africa, where HIV fuels TB cases,^6^ smoking is prevalent among people with HIV (PWH)^7^ and people with TB.^8,9^ The WHO and the International Union against Tuberculosis and Lung Disease (The Union) recommend providing smoking cessation interventions to people with TB,^10,11^ but they are often not included in programmes in LMICs, such as Botswana. While some studies have demonstrated the feasibility of smoking cessation interventions in TB clinics, additional information for implementation and scale-up is needed.^12^ TB treatment requires intensive follow-up and often includes support from community workers. Resource-limited settings may be able to leverage this structure and use TB diagnosis as a teachable moment^13^ to engage HIV+ smokers in tobacco cessation services.

We sought to identify key barriers and facilitators to integrating smoking cessation for PWH and TB into clinical care for these patients.

## METHODS

We employed a convergent parallel mixed-methods design.^14^ The quantitative component involved surveys administered to adults receiving TB treatment to characterise smokers receiving TB treatment in Gaborone, Botswana. The purposive sample included two groups: PWH receiving outpatient TB treatment and provider stakeholders identified by clinic leadership. The qualitative component aimed to understand the experience of smokers with TB engaging with providers about tobacco use, as well as their providers’ perspectives on addressing smoking. Interviews explored barriers and facilitators to implementing a programme. The integration of quantitative and qualitative components explored whether key themes were associated with participant characteristics to determine if the intervention would need to be tailored for subgroups of participants. All interviews were conducted in Setswana or English. This study was approved by the committees on human research of the University of Pennsylvania, the University of Botswana, and the Botswana Ministry of Health.

### Study sites and participants

We recruited patients and providers from 14 outpatient TB clinics in Greater Gaborone, Botswana. Enrolment occurred between 8 November 2023 and 22 August 2024. All participants provided written informed consent.

### Quantitative study procedure

Individuals aged ≥18 years on TB treatment were eligible. Data collected included demographics and medical and substance use history. Medical records were abstracted for TB- and HIV-related information and recorded in REDCap. The main quantitative variable of interest was smoking status at the time of TB diagnosis. The Alcohol Use Disorder Identification Test–Consumption (AUDIT-C) tool screened for unhealthy alcohol use,^15^ with binge drinking classified as ≥5 drinks per sitting and a cut-off score of ≥3 (women) or ≥ 4 (men) for unhealthy alcohol use.^16^ The Patient Health Questionnaire-9 (PHQ-9) was used to screen for depression with a cut-off score of ≥10, defining clinically significant depression symptoms.^17^

### Quantitative data analysis

All quantitative analyses were completed using STATA v.16 (StataCorp, College Station, Texas). A sample size of 120 participants was chosen based on an assumption of a smoking prevalence of up to 50%,^18^ which would provide a 95% confidence interval of <10%. In secondary bivariate analyses, we used Fisher’s exact test for categorical variables and the Wilcoxon rank-sum test for continuous variables to determine associations with smoking.

### Qualitative study procedure

Eligible patients were those with HIV who reported tobacco smoking at the time of TB diagnosis. Eligible providers were actively engaged in services at any level of TB treatment delivery. Semi-structured interview guides were developed using the Consolidated Framework for Implementation Research (CFIR)^19^ by the principal investigator, BL, with guidance from FB. Interviews with patients and providers were conducted one-on-one, in person, and audio-recorded by research assistants or BL. Interviewers explained that the goal was to understand how best to implement a behavioural intervention to support smoking cessation in TB clinics for PWH. Services would also include counselling for those experiencing symptoms of depression, given its high prevalence^20,21^ and its association with lower motivation to quit^22^ and relapse.^23^

### Qualitative data analysis

Audio recordings were transcribed and translated from Setswana to English, when necessary, by a professional translator, coded using an integrated approach, and analysed using NVivo 14 (Lumivero, Colorado). A priori codes based on CFIR constructs and grounded theory codes that emerged from close reading of the first five transcripts were used. Each code was defined, and decision rules for its application were recorded. Three research team members (BL, DA, and ON) independently coded responses and conducted meetings to compare readings of the data and assess interrater reliability. Team discussion refined the codebook and resolved discordances in coding. ON coded 100% and BL and DA coded 85% of transcripts. Key themes related to barriers and facilitators to implementing a smoking cessation programme in TB programmes were identified. These themes were then mapped onto CFIR constructs, which were matched with strategies outlined in the Expert Recommendations for Implementing Change (ERIC) framework,^24,25^ using the CFIR-ERIC matching tool.^26^ This process was confirmed by team consensus.

### Qualitative and quantitative data integration

We used a QUAL + quant data integration approach.^14^ Following the identification of key themes from the data related to implementing a smoking cessation programme, the quantitative data were used to identify the demographic characteristics of participants who expressed the identified themes.

### Ethics statement

This study was reviewed and approved by the institutional review boards of the University of Pennsylvania, the University of Botswana, and the Botswana Ministry of Health and Wellness. All study participants underwent written informed consent.

## RESULTS

A total of 168 individuals were screened in 14 TB clinics, with 137 individuals recruited to participate. Table 1 describes the demographics of individuals who reported smoking at the time of TB care initiation compared to individuals who did not. A smoking history was common, with 73/137 (53%) participants reporting ever smoking tobacco in the past. While 28/137 (20%) reported smoking at the time of TB diagnosis, only 14/28 (50%) of these individuals reported active smoking at the time of their interview. Of the 59/137 (43%) who reported being former smokers, 34/59 (58%) reported quitting within the last 6 months.

**Table 1.** Demographics of smokers versus non-smokers at the time of TB diagnosis.

Characteristics	Total, N = 137	Smoking at TB diagnosis, n = 28	Not smoking at TB diagnosis, n = 109	*P* value
Age in years; mean (SD)	39 (12)	38 (11)	39 (13)	0.887
Sex				
Male	94 (69%)	24 (86%)	70 (64%)	0.039
Female	43 (31%)	4 (14%)	39 (36%)	
Education (completed secondary school)	112 (82%)	23 (82%)	89 (82%)	>0.99
TB re-treatment	16 (12%)	3 (11%)	13 (12%)	>0.99
HIV				
Positive	73 (53%)	18 (64%)	55 (50%)	0.210
Negative	64 (47%)	10 (36%)	54 (50%)	
Diagnosis of non-communicable disease	23 (17%)	5 (18%)	18 (17%)	>0.99
Positive alcohol misuse screen				
Yes	55 (40%)	17 (61%)	38 (35%)	0.017
No	82 (60%)	11 (39%)	71 (65%)	
Prior marijuana use				<0.001
Yes	42 (31%)	18 (64%)	24 (22%)	
No	95 (69%)	10 (36%)	85 (78%)	
Clinically significant depression screen (PHQ ≥ 10)				
Yes	36 (26%)	10 (36%)	26 (24%)	0.232
No	101 (74%)	18 (64%)	83 (76%)	

PHQ = Patient Health Questionnaire.

The percentage listed in the overall column is the proportion of the categorical variable present in the overall population (137 participants). The percentages in the columns describing smoking status at the time of TB diagnosis are the proportion of individuals with the categorical variable within the specific smoking status group.

Overall, 70/137 (51%) reported alcohol use, with 29/70 (41%) reporting binge drinking at least weekly. Cannabis was the main illicit substance, with a third of participants reporting a history of prior use (Table 1). Only 12/137 (9%) reported use in the last 3 months, and most of these, 10/12 (83%), were daily users. Only 6/137 (4%) participants reported ever using inhalants, and 3/137 (2%) reported ever using amphetamines. There were no participants who reported prior cocaine or illicit opioid use.

Smokers at TB diagnosis were more likely to be male, have alcohol misuse, and have a history of prior cannabis use (Table 1). There was no association between HIV and smoking at TB diagnosis.

### Qualitative results

Twenty HIV/TB patients were interviewed (Table 2). Eight (40%) individuals reported smoking at the time of interview, while the rest reported they were smokers at the time of TB diagnosis and had since stopped. Nineteen providers were interviewed (Table 3). Six (32%) of the providers worked for non-governmental organisations (NGOs). We identified four key themes in the data, described below with representative quotes displayed in Table 4.

1. An approach using knowledge about the dangers of smoking to aid in tobacco cessation during TB care is necessary, but would likely be insufficient when used alone to facilitate smoking cessation.

**Table 2.** Demographics of patient stakeholders participating in qualitative interviews.

Demographics	Patient stakeholders
	Total = 20
Sex	
Male	17 (85%)
Female	3 (15%)
Age group	
26–44 years	15 (75%)
45–59 years	3 (15%)
≥60 years	2 (10%)
Education	
Primary education level	4 (20%)
Secondary education level	14 (70%)
Tertiary education level	2 (10%)
Unemployed	10 (50%)
Smoking status	
Current Smoker	8 (40%)
Smoking at TB diagnosis	12 (60%)
Depression PHQ-9 score	
Mild–moderate symptoms (≥1 and ≤9)	9 (45%)
Moderate–severe symptoms (≥10 and ≤27)	10 (50%)

PHQ-9 = Patient Health Questionnaire-9.

**Table 3.** Demographics of provider stakeholders.

Demographics	Provider stakeholders
	Total = 19
Sex	
Female	16 (84%)
Male	3 (16%)
Age group	
18–29 years	5 (26%)
30–44 years	9 (47%)
45–59 years	5 (26%)
# of years of health care experience	
<5	4 (21%)
5–14	8 (42%)
≥15	7 (37%)
Job titles	
Health care auxiliary	5
Community health worker	2
Research Assistant	2
Ministry of Health Officer	1
Nurse	3
Medical Doctor	1
Nurse Leadership	3
Ministry of Health Program Leadership	2

**Table 4.** Identified themes and representative quotes.

CFIR domain and theme	Representative quotes
Innovation (The thing being implemented): Knowledge is necessary but insufficient as a strategy.	‘At the time that I used to smoke, I could see that it was not good for me. I saw that it didn’t treat me well, it’s the one that made me get TB.’ (Patient, 19)
	‘We advise them, that when you have started TB treatment you should be pulling back if you are someone who smokes tobacco. You should cut back on your smoking, considering that we want you to get better. Smoking tobacco can also waste you, it kills your lungs and it can make the pills not to work the way that they are supposed to.’ (Provider 12, HCA)
	‘Yes, they sensitised me about smoking, but I continued to smoke til I understood little by little and eventually quit …. But even the way I quit, it was because I felt the signs in my body, that I wasn’t feeling very well, my chest was painful … and I thought that maybe it’s the thing I have been warned against, so I decided to quit.’ (Patient 14)
Individuals (Features and beliefs of the people involved): Providers lack training and are uncomfortable with interacting with TB patients.	‘There is no training that was ever done, so I can say they are incompetent to give that information.’ (Provider 11, Clinic Leadership)
	‘They were telling me you should quit smoking, but they …. (were) not telling me how to quit or giving me ideas or pamphlets or whatever or tell me what tobacco would do to me ….’ (Patient 20)
	‘When we deal with TB, what I have seen is that even though people are nurses, they are still also afraid to associate themselves with the patients. The way I have realised, as we have been going around with the nurses, it’s like they also have not accepted how to deal with TB patients.’ (Provider 15, Research Assistant)
Inner setting (Characteristics of the clinic setting): TB care system is viewed positively but lacks the capacity for a cessation programme.	‘I’ve been well-taken care of. My providers have been very helpful, useful, and patient with me.’ (Patient 10)
	‘I take it that the program could be very helpful, it is a good program that can help a lot of people who want to stop smoking and those who have a lot of depression.’ (Provider 3, CHW)
	‘I don’t know if it would work, if it would be isolated to TB only. Because we know that it’s … You know it’s broad. Everybody uses tobacco. … I don’t think it would be ok for us to just isolate it to TB ....’ (Provider 19, MOH Leadership)
	‘The thing is, right now we cannot really relax because we are looking at the fact that we don’t want to keep them for long among general patients. If there was a special place where we could see them, where one can have some time with them without looking at assisting them to come out from among the public like is currently happening.’ (Provider 5, Nurse)
	‘In this clinic, it’s the workload that can affect them, because like I have said, one person is expected to cover three areas in a day. Even at a personal level, where you had wanted to really talk to people, you end up just doing the bare minimum and taking shortcuts so that you can accommodate others. So, our workload at the moment can hinder us from properly implementing the program the way we would like.’ (Provider 5, Nurse)
Outer setting (Characteristics of extra-institutional factors affecting implementation): Leveraging NGOs would be beneficial but may have negative long-term effects on longevity of a programme.	‘[The] government can’t work alone, you see, they are always non-governmental organisations that are there to help in this implementation like organisations like BUMMHI are available in clinic to assist in these programs like TB and I think I mentioned for substance abuse maybe if they can be included because most of the patients come to government clinics. They [NGOs] can come and help because government alone can’t do it.’ (Provider 2, HCA)
	‘Yes, normally they [NGOs] do come with very good initiatives ... like the one on depression. Maybe they can come to assist, and then the problem is when they go, it means that this program will ... collapse ….’ (Provider 11, Clinic Leadership)
	‘The chief leaders know the people. They know the behaviors of the people, and they also know what helps that community. They also know what helps that community, so if an intervention is going to work in their community, they will know and they will tell you, and if it’s not going to work, they will also tell you that this thing you are coming with is not going to work for us.’ (Provider 19, Program Leadership)

CFIR = Consolidated Framework for Implementation Research; CHW = community health worker; HCA = health care auxiliary; MOH = Ministry of Health; NGO = non-governmental organisation.

There was a good understanding among both patients and providers that smoking causes damage to the lungs, as does TB infection, and tobacco cessation, particularly during treatment, is a difficult but important part of the management of TB infection to prevent further complications and facilitate successful treatment. The role of addiction-informed counselling was not raised by providers. Even when the difficulty with smoking cessation or addiction was acknowledged, providers emphasised education on the dangers of smoking as the only way to support smoking cessation. Participants who were able to quit smoking cited free will and adverse symptoms as the mechanism underpinning their ultimate quitting.

2. Providers feel inadequate in providing smoking cessation services and are apprehensive about interacting with TB patients.

Providers described a lack of formal training or resources for smoking cessation services, while patients described being told to quit smoking but not being given the tools to do so. In addition to a lack of skills to provide smoking cessation services, providers described observing among staff a general discomfort or reluctance with caring for TB patients due to personal infection risk.

3. Embedding smoking cessation in the TB clinic would be facilitated by a positive view of the TB care setting and a perceived need for smoking cessation services, but it will require additional clinic resources and cannot just be limited to specific populations.

Patients describe the current TB care setting as generally supportive, reporting positive experiences and close follow-up with providers during treatment. Both providers and patients expressed enthusiasm about a potential intervention for smoking cessation, with providers acknowledging that many of their patients used tobacco, and resources to assist with smoking cessation would be welcome. Despite the enthusiasm for a smoking cessation programme, providers were concerned that the proposed services would only be available to individuals with TB. Many expressed a widespread need for cessation services in the clinics. They felt that concentrating an intervention on a particular population, like people with TB and HIV, would leave out other patients who could also benefit. Providers mentioned several clinic constraints that would need to be addressed to support the implementation of an intervention. These concerns included a lack of space in the clinic, particularly a lack of infrastructure to safely provide services to TB patients without putting other patients and providers in the clinic at risk of infection. To help address the lack of space in clinics and ensure that implementers could deliver a new intervention, providers suggested using temporary structures such as open-air shelters and portable cabins to create additional workspace. Providers also mentioned the existing clinic workload and staffing shortages as significant barriers to implementing smoking cessation services in the clinic, given many competing priorities. While they stated the need for additional personnel to be hired to deliver the proposed intervention, they also noted that training existing staff in the intervention would provide them with the tools and motivation to be involved in implementation.

4. Leveraging non-governmental implementation partners would be an overall asset to a tobacco cessation programme, but may also have the potential to impede its longevity if there is over-reliance on external implementers.

Providers expressed the need to engage external organisational stakeholders, such as NGOs, to support a cessation programme, including leveraging their expertise to help train providers. While both governmental and NGO providers described the need to involve NGOs as implementation partners, some government providers described past challenges with new interventions that come to the clinic heavily supported by implementation partners, which work well for a period but ultimately fail when partners hand over the work to the clinic staff. Apart from NGOs, providers also recommended engaging other community stakeholders external to the clinic, such as community leaders and local churches, to support the development, engagement, and dissemination of a tobacco cessation programme.

### Strategies for implementation identified using CFIR-ERIC matching tool

Identified themes were matched onto CFIR constructs, and the CFIR-ERIC matching tool identified several implementation strategies that could be used to leverage facilitators and address barriers to implementation. These are displayed in Table 5.

**Table 5.** Key themes mapped onto CFIR constructs and ERIC implementation strategies.

Themes	CFIR construct	ERIC implementation strategy
Education on the dangers of tobacco use to aid in cessation is necessary but insufficient when used alone		
1.1 Patients understand the importance of tobacco cessation, but education is not enough to make them quit.	Innovation: relative advantage	Engage consumers (prepare patients to participate, educate patients); Develop stakeholder relationships (identify/prepare champions; import local leaders)
1.2 Providers stated patient education as the only available strategy to facilitate smoking cessation	Innovation: relative advantage	Develop stakeholder relationships (identify/prepare champions; import local leaders); Provide technical assistance; Promote network weaving; Fund and contract for the clinical innovation
Providers lack training and are uncomfortable with interacting with TB patients		
2.1 Providers require training on smoking cessation services	Individual characteristics: capability	Provide interactive assistance (clinical supervision, training, facilitation, champions)
2.2 Providers apprehensive about infection risk involved in interacting with TB patients	Individual characteristics: motivation	Change infrastructure (mandate change; leadership declaration); Support clinicians (revise professional roles, restructure staffing, change policies)
TB care system is viewed positively but lacks the capacity for a cessation programme		
3.1 Clinic providers have strong rapport with their patients and patients feel well cared for in the TB care setting.	Inner setting: mission alignment	Build a coalition; Facilitation; Involve patients and family members; Prepare patients to be active participants
3.2 Need for additional infrastructure like staff and buildings to support additional services	Inner setting: structural characteristics	Change infrastructure; Promote adaptability; Promote network weaving; Identify and Prepare champions; Identify early adopters; Build a coalition
Leveraging NGOs would be beneficial but may have negative long-term effects on longevity of a programme		
4.1 NGOs play an important role in patient care and will be important to leverage to provide additional services	Outer setting: partnerships and connections	Build a coalition; Capture and share local knowledge; Create a learning collaborative; Develop resource sharing agreements; Develop academic partnership; Promote network weaving

CFIR = Consolidated Framework for Implementation Research; ERIC = Expert Recommendations for Implementing Change.

### Qualitative and quantitative data integration

There was consistency between the qualitative and quantitative data in theme 1, with women more likely than men to report that they did not receive education about smoking during their health care visits. During qualitative interviews, 2/3 (67%) women reported that their ongoing tobacco use had never been addressed while receiving TB treatment, compared to 5/17 (29%) of men. On review of the larger survey cohort, 3/4 (75%) women and 13/24 (54%) men smoking at TB diagnosis reported they had never received any advice on smoking cessation from a health care provider. Our analysis did not uncover any other patterns in demographics among the identified themes.

## DISCUSSION

Our survey found a smoking prevalence among TB patients in Botswana similar to prior reports.^8,27,28^ This finding is likely an underestimation of individuals who would benefit from smoking cessation services, as more than half of former smokers had quit in the last 6 months, raising the possibility that TB-related symptoms influenced their tobacco cessation, making them high risk for relapse as symptoms abate with treatment.^29,30^ A quarter of TB patients, including over a third of smokers receiving TB treatment, reported clinically significant depression symptoms, similar to another study that measured depression symptoms in smokers with TB in Botswana,^8^ and consistent with other TB studies.^31^ This finding emphasises a need to ensure the availability of mental health–related services within the TB care setting, a recommendation supported by the WHO.^32^

This study was not powered to detect differences between groups, but in an exploratory analysis, we found that a higher proportion of smokers at TB diagnosis were PWH. This difference was most prominent in men. While not statistically significant, the magnitude of the difference was consistent with the literature.^7^ Interestingly, women were more likely to report that they had not received advice on smoking cessation during their TB treatment. This could be related to bias from health care providers who may see women as unlikely to smoke and therefore not screen for tobacco use. A universal screening approach would help address this bias.

This study identified several important considerations when planning for the implementation of smoking cessation services within the TB care setting in Botswana and similarly resourced settings. Smokers with TB expressed great interest in smoking cessation services and found the TB care setting to be a supportive environment. While this finding could be leveraged to introduce services, health care stakeholders were averse to excluding non-TB patients who may benefit from such services. Given the need for tobacco cessation services beyond the TB and HIV care setting, programmes could consider coupling cessation services with established non-communicable disease programmes like hypertension and diabetes, with an emphasis on ensuring their inclusion in programmes that are integrated into HIV clinics, an approach that has the potential to be successful for comprehensive care delivery for PWH.^33^ This approach, which may need to be tailored based on country-specific needs and resources, would make services available to the broader public and simultaneously ensure optimal delivery for those with HIV or TB.

Several ERIC implementation strategies were identified that could inform the development of a potential implementation science innovation for testing. These included an emphasis on engaging stakeholders and building a network of infrastructure by using strategies such as intervention champions and identifying funding and technical support for the innovation.

This study had several limitations to consider. It was conducted in a single district; however, we were able to interview a diverse range of stakeholders. Additionally, the results may not be generalisable to more rural clinics with fewer resources available, although we did include perspectives from both urban and peri-urban clinics. Social desirability bias also likely impacted self-reporting of smoking status. Smoking is likely more prevalent than we found, and therefore an even more pressing issue.

## CONCLUSION

These findings highlight critical considerations for tobacco cessation services in resource-limited settings, informing the development of implementation strategies for cessation interventions in this context. Given the widespread need for tobacco cessation services, LMICs that do not have existing services within their health system may need to first prioritise an implementation approach available for all smokers, not just those with HIV or TB.

## References

[1] World Health Organization. Global tuberculosis report 2021. Geneva: WHO, 2021.

[2] Samet JM. Tobacco smoking: the leading cause of preventable disease worldwide. Thorac Surg Clin. 2013;23(2):103-112. 23566962 10.1016/j.thorsurg.2013.01.009

[3] Lin H-H, Ezzati M, Murray M. Tobacco smoke, indoor air pollution and tuberculosis: a systematic review and meta-analysis. PLoS Med. 2007;4(1):e20. 17227135 10.1371/journal.pmed.0040020 PMC1769410

[4] Burusie A, Effect of smoking on tuberculosis treatment outcomes: a systematic review and meta-analysis. PLoS One. 2020;15(9):e0239333. 32941508 10.1371/journal.pone.0239333 PMC7498109

[5] Huang CC, Cigarette smoking among tuberculosis patients increases risk of transmission to child contacts. Int J Tuberc Lung Dis. 2014;18(11):1285-1291. 25299859 10.5588/ijtld.14.0309

[6] Kwan CK, Ernst JD. HIV and tuberculosis: a deadly human syndemic. Clin Microbiol Rev. 2011;24(2):351-376. 21482729 10.1128/CMR.00042-10 PMC3122491

[7] Murphy JD, Liu B, Parascandola M. Smoking and HIV in Sub-Saharan Africa: a 25-country analysis of the demographic health surveys. Nicotine Tob Res. 2019;21(8):1093-1102. 30165688 10.1093/ntr/nty176 PMC6941705

[8] Jones-Patten A, Depression, anxiety, and cigarette smoking among patients with tuberculosis. Clin Nurs Res. 2022;32(1):22-28. 36285635 10.1177/10547738221132096 PMC9749560

[9] Lam C, Prevalence of tobacco smoking in adults with tuberculosis in South Africa. Int J Tuberc Lung Dis. 2013;17(10):1354-1357. 23827797 10.5588/ijtld.13.0016

[10] Bissell K, Smoking cessation and smokefree environments for tuberculosis patients. 2nd ed. Paris, France: International Union Against Tuberculosis and Lung Disease, 2010. https://theunion.org/sites/default/files/2020-08/pub_smokingcessation_eng.pdf.

[11] World Health Organization. A WHO/The Union monograph on TB and tobacco control: joining efforts to control two related global epidemics. Geneva: WHO, 2007. https://theunion.org/sites/default/files/2020-08/9789241596220_eng.pdf.

[12] Whitehouse E, A systematic review of the effectiveness of smoking cessation interventions among patients with tuberculosis. Public Health Action. 2018;8(2):37-49. 29946519 10.5588/pha.18.0006 PMC6012961

[13] Elsey H, Predictors of cessation in smokers suspected of TB: Secondary analysis of data from a cluster randomized controlled trial. Drug Alcohol Depend. 2015;155:128-133. 26297296 10.1016/j.drugalcdep.2015.08.002

[14] Palinkas LA, Mixed method designs in implementation research. Adm Policy Ment Health. 2011;38(1):44-53. 20967495 10.1007/s10488-010-0314-z PMC3025112

[15] Bush K, The AUDIT alcohol consumption questions (AUDIT-C): an effective brief screening test for problem drinking. Ambulatory care quality improvement project (ACQUIP). Alcohol use disorders identification test. Arch Intern Med. 1998;158(16):1789-1795. 9738608 10.1001/archinte.158.16.1789

[16] Bradley KA, AUDIT-C as a brief screen for alcohol misuse in primary care. Alcohol Clin Exp Res. 2007;31(7):1208-1217. 17451397 10.1111/j.1530-0277.2007.00403.x

[17] Levis B, Accuracy of patient health questionnaire-9 (PHQ-9) for screening to detect major depression: individual participant data meta-analysis. BMJ. 2019;365:l1476. 30967483 10.1136/bmj.l1476 PMC6454318

[18] Mosepele M, Cholesterol screening and statin prescription is low among HIV-infected patients on protease-inhibitor regimens in Botswana. Open AIDS J. 2017;11:45-51. 28839514 10.2174/1874613601711010045 PMC5543697

[19] Damschroder LJ, The updated consolidated framework for implementation research based on user feedback. Implement Sci. 2022;17(1):75. 36309746 10.1186/s13012-022-01245-0 PMC9617234

[20] Gupta R, Depression and HIV in Botswana: a population-based study on gender-specific socioeconomic and behavioral correlates. PLoS One. 2010;5(12):e14252. 21170384 10.1371/journal.pone.0014252 PMC2999532

[21] Molebatsi K, Depression and delayed tuberculosis treatment initiation among newly diagnosed patients in Botswana. Glob Public Health. 2021;16(7):1088-1098. 32991275 10.1080/17441692.2020.1826049 PMC8005506

[22] Burkhalter JE, Tobacco use and readiness to quit smoking in low-income HIV-infected persons. Nicotine Tob Res. 2005;7(4):511-522. 16085522 10.1080/14622200500186064

[23] Zvolensky MJ, Quit-attempt history: relation to current levels of emotional vulnerability among adult cigarette users. J Stud Alcohol Drugs. 2009;70(4):551-554. 19515295 10.15288/jsad.2009.70.551

[24] Powell BJ, A refined compilation of implementation strategies: results from the expert recommendations for implementing change (ERIC) project. Implement Sci. 2015;10:21. 25889199 10.1186/s13012-015-0209-1 PMC4328074

[25] Waltz TJ, Use of concept mapping to characterize relationships among implementation strategies and assess their feasibility and importance: results from the expert recommendations for implementing change (ERIC) study. Implement Sci. 2015;10:109. 26249843 10.1186/s13012-015-0295-0 PMC4527340

[26] Waltz TJ, Choosing implementation strategies to address contextual barriers: diversity in recommendations and future directions. Implement Sci. 2019;14(1):42. 31036028 10.1186/s13012-019-0892-4 PMC6489173

[27] Arnold Sejie G, Mahomed O. Predictors of tuberculosis outcomes amongst drug sensitive patients in Boteti sub-district, Botswana, 2015-2017. Indian J Tuberc. 2020;67(1):79-86. 32192622 10.1016/j.ijtb.2019.04.014

[28] Zetola NM, Population-based geospatial and molecular epidemiologic study of tuberculosis transmission dynamics, Botswana, 2012-2016. Emerg Infect Dis. 2021;27(3):835-844. 33622470 10.3201/eid2703.203840 PMC7920683

[29] Ng N, Smoking behavior among former tuberculosis patients in Indonesia: intervention is needed. Int J Tuberc Lung Dis. 2008;12(5):567-572. 18419894

[30] Pradeepkumar AS, Thankappan KR, Nichter M. Smoking among tuberculosis patients in Kerala, India: proactive cessation efforts are urgently needed. Int J Tuberc Lung Dis. 2008;12(10):1139-1145. 18812043

[31] Duko B, Bedaso A, Ayano G. The prevalence of depression among patients with tuberculosis: a systematic review and meta-analysis. Ann Gen Psychiatry. 2020;19:30. 32419837 10.1186/s12991-020-00281-8 PMC7206806

[32] World Health Organization. Implementing the end TB strategy. Geneva: WHO, 2016.

[33] Chireshe R, Manyangadze T, Naidoo K. Integrated chronic care models for people with comorbid of HIV and non-communicable diseases in Sub-Saharan Africa: a scoping review. PLoS One. 2024;19(3):e0299904. 38489252 10.1371/journal.pone.0299904 PMC10942093

